# Characteristic abnormalities in cerebrospinal fluid biochemistry in children with cerebral malaria compared to viral encephalitis

**DOI:** 10.1186/1743-8454-3-8

**Published:** 2006-06-09

**Authors:** SR Jakka, S Veena, RM Atmakuri, M Eisenhut

**Affiliations:** 1 Whiston General Hospital, Whiston, Warrington Road, L355DR, UK; 2 Government General Hospital, Kakinada, India; 3 Institute of Child Health, University of Liverpool, Eaton Road, Liverpool, L12 2AP, UK

## Abstract

**Background:**

In developing countries where *Plasmodium falciparum *malaria is endemic, viral encephalitis and cerebral malaria are found in the same population, and parasitemia with *Plasmodium falciparum *is common in asymptomatic children. The objective of this study was to investigate the cerebrospinal fluid (CSF) biochemistry in children with cerebral malaria compared to those with presumed viral encephalitis.

**Methods:**

We studied the following CSF parameters: cell count, glucose, protein, lactic dehydrogenase (LDH) and adenosine deaminase (ADA) levels, in children with cerebral malaria, with presumed viral encephalitis, and in control subjects who had a lumbar puncture after a febrile convulsion with postictal coma.

**Results:**

We recruited 12 children with cerebral malaria, 14 children with presumed viral encephalitis and 20 controls prospectively, over 2 years in the Government General Hospital in Kakinada, India. Patients with cerebral malaria had significantly lower CSF glucose, and higher protein, LDH, CSF/blood LDH ratio and CSF ADA levels but a lower CSF/serum ADA ratio compared to controls (*p *< 0.01). Patients with cerebral malaria had lower CSF white cell count, glucose, protein, LDH levels and CSF/serum ADA ratio compared to patients with presumed viral encephalitis. CSF/serum ADA ratio was lower in patients with cerebral malaria due to the fact that serum ADA levels were significantly higher in patients with cerebral malaria compared to the other two groups. A CSF/serum ADA ratio of <0.38 and a CSF glucose level of <3.4 mmol/l were selected as the cut-off values with the highest sensitivities and specificities for comparing the two conditions.

**Conclusion:**

CSF/serum ADA ratio and CSF glucose levels were the best discriminators of cerebral malaria from presumed viral encephalitis in our study. Further studies are needed to explore their usefulness in epidemiological studies.

## Background

Previous studies have compared cerebrospinal fluid (CSF) features of bacterial meningitis and cerebral malaria [1,2]. This distinction is in most cases not difficult, because the majority of patients with bacterial meningitis have marked CSF pleocytosis, in contrast to cerebral malaria where this is uncommon. More difficult is the distinction of cerebral malaria from viral encephalitis on clinical grounds or from a CSF cell count. Rapid virological diagnostic methods are not available in most developing countries. With the benefit of acyclovir in herpes encephalitis for prevention of death or neurological sequelae [3], and the need to recognize the presence of possible arbovirus encephalitis with its public health implications [4], it has become more important to differentiate viral encephalitis from cerebral malaria. The objective of our study was, therefore, to investigate the distinguishing features of a variety of CSF parameters in patients with cerebral malaria as opposed to those with presumed viral encephalitis. We have chosen for our investigation CSF cell count, glucose and protein levels, together with CSF and serum lactic dehydrogenase (LDH) and adenosine deaminase (ADA). Previous investigations have found that these parameters may be useful in discrimination of infectious diseases of the central nervous system such as meningitis, encephalitis and cerebral malaria [5,6]. Lactic dehydrogenase is an intracellular enzyme that is released from damaged cells. Its level in the CSF reflects the degree of damage to cells in the central nervous system. CSF- adenosine deaminase, an enzyme mainly produced by developing immature T-lymphocytes, is increased in the body fluids of patients with conditions associated with stimulation of cellular immunity and was evaluated in this study for its use in differentiation of parasitic and viral infection of the brain.

## Methods

### Patients

In a 2-year prospective study at Government General hospital in Kakinada, India we recruited with ethical approval and informed consent by parents or guardians, children with a clinical diagnosis of presumed viral encephalitis, cerebral malaria and controls. The definition of presumed viral encephalitis was a reduced level of consciousness and pyrexia that could not be explained by a metabolic abnormality, dehydration or shock, negative blood and CSF cultures, and a negative blood slide for malaria parasites with recovery without antibiotics or lack of response to broad-spectrum antibiotics [7]. Cerebral malaria was defined as coma and pyrexia with a positive thick film for asexual *P. falciparum *blood stages and no other identified cause of an encephalopathy following the WHO definition [8]. We recruited control subjects from a population of patients who had lumbar punctures to exclude meningitis in the context of reduced consciousness following febrile convulsions and who recovered from the postictal state without further signs of central nervous system illness or septicaemia.

### Sample collection and analysis

CSF was obtained by lumbar puncture as soon as possible after admission if there were no contraindications, and after informed consent by the parents. CSF cell count, glucose, protein and CSF and serum LDH levels were determined by standard methods as in a previous study published by this group [9]. ADA levels were determined using the Berthelot reaction, through the ammonia released when adenosine is broken down to inosine. After incubation of plasma or CSF with a buffered solution of adenosine, the ammonia is reacted with a Berthelot reagent to form a blue colour, which is proportional to the amount of enzyme activity [10].

### Data analysis

For multiple comparisons of data with an approximately normal distribution the one way analysis of variance (ANOVA) was used. Tukey's HSD test was used for post hoc analysis of these data. For non-parametric data the Kruskal-Wallis test was used. The statistical method used for univariate comparisons of continuous data was the Mann-Whitney test, and for categorical data the chi-square test. For determination of a cutoff value for a CSF parameter for possible discrimination between presumed viral encephalitis and cerebral malaria, the co-ordinate of a receiver operating characteristic (ROC) curve indicating the greatest sensitivity and specificity was chosen (for explanation of the statistical methodology see [11]). The ROC curve is a computer-generated curve of data from the malaria and viral encephalitis patients with sensitivity on the vertical axis plotted against 1-specificity (1-true negative rate) on the horizontal axis. Sensitivity is the true positive rate in percent and it was calculated from the ratio of the number of true positive over the sum of true positive and false negative patients. True positive in the context of this study, was the presence of cerebral malaria in any patient below a certain value of a parameter, the 'cut-off'. Specificity is the true negative rate in percent and it was calculated as the ratio of the number of true negative over the sum of false positive and true negative patients. True negative meant the patient has presumed viral encephalitis if the parameter is above the "cut-off" chosen (Table 2). The positive predictive value is the post-test probability of a positive test and negative predictive value the post-test probability of a negative test [12]. The positive predictive value is calculated as the ratio of the number of true positive over the sum of true positive and false positive patients. The negative predictive value is the ratio of the number of true negative over the sum of false negative and true negative patients. The probability for the positive predictive value was the probability of having cerebral malaria in any patient and for the negative predictive value, the probability of having presumed viral encephalitis. A *p*-value < 0.05 was taken as indicator for a statistically significant difference. Statistical calculations were performed with SPSS version 13.0 and Epi Info 6.04b (CDC, Atlanta). Confidence intervals for parameters were calculated by Confidence Interval Analysis software.

## Results

**Figure 1:**
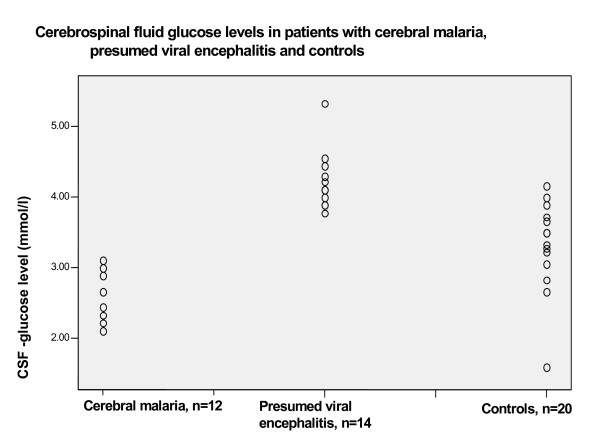
Scatter plot of CSF glucose levels in patients with cerebral malaria, presumed viral encephalitis and controls.

**Figure 2:**
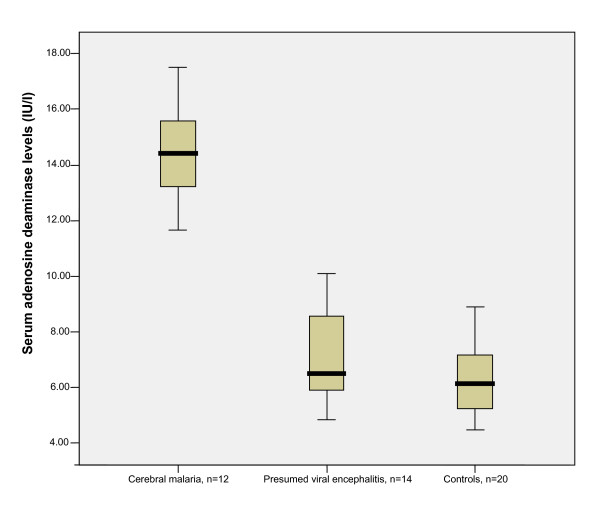
Box plot of serum adenosine deaminase levels in patients with cerebral malaria, presumed viral encephalitis and controls. The line inside the box represents the median, the box the quartiles, and the whiskers the extreme values.

46 children were recruited over a 2-year period. Twelve patients had cerebral malaria, 14 patients presumed viral encephalitis and 20 patients were controls. Demographic and laboratory parameters including LDH and ADA CSF/serum ratios are listed in Table 1. There was no significant difference in age between patients with cerebral malaria and presumed viral encephalitis, although both groups were both significantly older than controls. The CSF white cell count was significantly higher in the group with presumed viral encephalitis compared to patients with cerebral malaria or controls. The mean CSF glucose level was significantly lower in patients with cerebral malaria compared to the other two groups and the range for glucose levels in patients with cerebral malaria did not overlap with that for patients with presumed viral encephalitis (Figure 1). Patients with cerebral malaria had significantly higher CSF protein, LDH and ADA levels and CSF/serum LDH ratio but a lower CSF/serum ADA ratio, compared to controls (Table 1). Patients with cerebral malaria had significantly lower CSF glucose, protein and LDH levels and CSF/serum ADA ratios compared to patients with presumed viral encephalitis (Table 1). Patients with cerebral malaria had a significantly higher serum ADA level compared to patients with presumed viral encephalitis and to controls (Mann-Whitney test, p < 0.05, Figure 2). Serum ADA levels in patients with presumed viral encephalitis were not significantly different from controls (*p *> 0.05). Serum LDH levels were not significantly different between groups (*p *> 0.05). However, CSF/Serum LDH ratios for both patients with cerebral malaria and presumed viral encephalitis were significantly higher than controls.

**Table 1:** Comparison of demographic and cerebrospinal fluid parameters between patients with cerebral malaria, presumed viral encephalitis and controls.

	Cerebral malaria n = 12	Presumed viral encephalitis n = 14	Controls n = 20	*p*-value for multiple comparisons
Age (years)	7.0 (2.7)	7.0 (3.5)	1.7 (1.2)	<0.01^a^
Gender (male)	7	6	9	ns^b^
CSF-parameters:				
White cells (cells/μl)^c^	0 (0–3)	4 (0–9)	0 (0–4)	<0.01^d^
Glucose (mmol/l)	2.7 (0.3)	4.2 (0.4)	3.3 (0.6)	<0.01^a^
				
Protein (g/l)	0.4 (0.04)	0.5 (0.06)	0.3 (0.10)	<0.01^a^
CSF-ADA (IU/l)	4.6 (0.9)	3.8 (1.0)	3.3 (0.8)	<0.01^a^
				
Serum ADA (IU/l)^c^	14.4 (11.6–17.5)	6.5 (4.8–10.1)	6.1 (4.4–8.9)	<0.01^d^
Ratio CSF/serum ADA	0.31 (0.05)	0.54 (0.11)	0.53 (0.13)	<0.01^a^
CSF-LDH (IU/ml)	20.7 (2.8)	26.5 (5.8)	15.9 (3.8)	<0.01^a^
				
Serum LDH (IU/ml)	214.1 (25.7)	247.5 (46.8)	244.0 (57.7)	ns ^a^
Ratio CSF/serum LDH	0.09 (0.02)	0.11 (0.02)	0.07 (0.01)	<0.01^a^

LDH = Lactic dehydrogenase, ADA = Adenosine deaminase. Results where applicable are given as mean (SD). ^a ^Analysis of variance: Post hoc analysis results with p < 0.05 (Tukey HSD test): *Age*: Cerebral malaria and presumed viral encephalitis versus controls. *CSF white cell count*: Cerebral malaria versus presumed viral encephalitis and presumed viral encephalitis versus controls. *Glucose and protein*: Each group versus the 2 other groups. CSF-*ADA*: Cerebral malaria versus controls. *Serum ADA*: Cerebral malaria versus the 2 other groups. *Ratio CSF/serum ADA*: Cerebral malaria versus the two 2 groups. *CSF-LDH*: Each group versus the other 2 groups. *Ratio CSF/serum LDH*: Cerebral malaria and presumed viral encephalitis versus controls. ^b ^ns = not significant by chi-square test. ^c ^Median and range. ^d ^Kruskal-Wallis test.

**Table 2:** Cerebrospinal fluid parameters as discriminators between cerebral malaria and presumed viral encephalitis

Cut-off	Sensitivity^1 ^(%)	Specificity^2 ^(%)	Negative predictive Value^3 ^(%)	Positive predictive Value^4 ^(%)
	(95% CI)	(95% CI)	(95% CI)	(95% CI)
CSF white cell count <4 cells/microl	100 (76–100)	57 (32–78)	100 (67–100)	66 (43–83)
CSF protein <0.43 g/l	92 (64–98)	78 (52–92)	91 (64–98)	78 (52–92)
CSF glucose <3.4 mmol/l	100 (78–100)	100 (75–100)	100 (75–100)	100 (78–100)
Ratio CSF/serum ADA <0.385	91 (64–98)	100 (78–100)	93 (70–100)	100 (74–100)
				
CSF LDH < 22.5 IU/ml	75 (46–91)	71 (44–88)	76 (49–92)	69 (42–87)

^1^Sensitivity is the true positive rate in percent. It is calculated as the ratio of the number of true positive over the sum of true positive and false negative patients. True positive meant in the context of this study the presence of cerebral malaria in a group of patients with presumed viral encephalitis or cerebral malaria below a certain value of a parameter, the "cut-off". ^2^Specificity is the true negative rate in percent. It is calculated as the ratio of the number of true negative over the sum of false positive and true negative patients. True negative meant the patient has presumed viral encephalitis if the parameter is above the "cut-off" chosen. ^3^The negative predictive value is the ratio of the number of true negative over the sum of false negative and true negative patients. Positive predictive value is the post-test probability of a positive test and negative predictive value the post-test probability of a negative test. ^4^The positive predictive value is calculated as the ratio of the number of true positive over the sum of true positive and false positive patients.

For markers with a significant difference between cerebral malaria and the presumed viral encephalitis groups, we determined cut-off values of CSF parameters with maximum sensitivities and specificities and positive and negative predictive values. The best cut-off values taken from the co-ordinates of the ROC curve (see methods section) below which cerebral malaria was more likely than presumed viral encephalitis were: CSF white cell count, 4 cells/μl; CSF protein level, 0.43 g/l; CSF glucose, 3.4 mmol/l; CSF LDH, 22.5 IU/ml; CSF/serum ADA ratio, 0.385 (Table 2).

CSF white cell count, protein and glucose levels and CSF/serum ADA ratio below these cut-off values indicated cerebral malaria with a sensitivity of > 90%. This resulted in the probability of having presumed viral encephalitis in our group of children with cerebral malaria or viral encephalitis (or negative predictive value for having cerebral malaria) being above 90% if the values of these parameters were above this cut-off. Specificities for the parameters were 100% for CSF glucose and 100% for CSF/serum ADA ratio and better than for other parameters, i.e. presumed viral encephalitis was always present in any patient with a value above the cut-off. This resulted in a probability (positive predictive value) of 100% of having cerebral malaria in children with parameters below this cut-off (Table 2).

## Discussion

This is to our knowledge the first study to investigate differences in CSF parameters between presumed viral encephalitis and cerebral malaria. Asymptomatic parasitaemia with *Plasmodium falciparum *is common in regions hyperendemic for this parasite. In many of these areas, particularly in South East Asia, viral encephalitis is also a public health problem. It is therefore important to investigate discriminating CSF features between encephalitis and cerebral malaria. Our study is the most detailed study on CSF parameters in cerebral malaria reported so far. We could not, however, exclude the possibility that patients labeled as having cerebral malaria had a combination of encephalitis and parasitaemia with *P. falciparum*, but the lack of white cells in their cerebrospinal fluid make this possibility seem very unlikely.

### CSF-glucose

A CSF glucose level below 3.4 mmol/l was the best discriminator of cerebral malaria from presumed viral encephalitis. This was partly due to the fact that in cerebral malaria CSF glucose levels were below normal range. Low CSF glucose is a well-known phenomenon in cerebral malaria and CSF glucose has been found to be lower in patients with fatal cerebral malaria as compared to survivors [13]. However, the low CSF glucose may be partly due to low plasma glucose levels not measured in this study, but previously found in falciparum malaria [14]. The previous study found CSF glucose levels of up to 7 mmol/l in patients with cerebral malaria and mean levels of 4.3 mmol/l in survivors, which indicates that a discriminator of 3.4 mmol/l between cerebral malaria and encephalitis may not be universally applicable, but dependent on disease severity [13]. Febrile convulsions were an indication for lumbar puncture in the control patients. Consequently the control patients in our study were significantly younger than the disease cases, due to the young age at which febrile convulsions usually occur. CSF glucose levels are independent of age above 2 months of age with lower levels found in infants below this age [15]. All the children in our study were older than 3 months and therefore the observed differences were not age related. Elevated CSF glucose has been described in cases of viral encephalitis [16] and this may have contributed to the significant difference found here between the two groups.

### CSF-protein

Previous studies with data on CSF in cerebral malaria also found increased total protein levels [17,18]. Our result of increased cerebrospinal fluid protein levels in presumed viral encephalitis has also been found in patients with herpes encephalitis [19,20].

### Adenosine deaminase

We found that a CSF/serum ADA ratio of <0.38 was the best discriminator of cerebral malaria from presumed viral encephalitis. CSF ADA levels were measured in 3 patients with cerebral malaria previously, and found to have a mean (SD) of 6.6 (1.03) IU/l [5], slightly higher than the levels in our study (4.6 (0.9) IU/l). There has been no previous investigation of serum ADA levels in *P. falciparum *malaria but serum ADA levels were found to be elevated to more than twice the level of controls in a previous study on patients with *Plasmodium vivax *malaria [21]. CSF ADA levels in a previous study containing data on 10 patients with viral encephalitis were found to have a mean (SD) of 6.15 (2.93) [6], which is higher than the levels reported here (3.8 (1.0) IU/l). Although the etiological agent of the presumed viral encephalitis was not determined, it was most likely to be a heterogeneous group over the 2-year period during which the patients were recruited [22].

### Lactic dehydrogenase

Mean CSF LDH levels of 26.5 U/ml in our patients with encephalitis were similar to the means of 22.6 and 22.3 U/ml found in two previous studies, respectively, [23,24]. Our controls had a slightly lower mean CSF LDH level than the 20 controls (adults and children without neurological illness) of a previous study with a mean level of 21.8 U/ml [24]. There is to our knowledge no previous study on CSF LDH levels in cerebral malaria.

CSF lactate, which is characteristically elevated in cerebral malaria [13], is another parameter for future studies looking at discriminating features between cerebral malaria and encephalitis.

Patients with clinical features of encephalitis and a positive malaria blood slide should still be treated as suffering from cerebral malaria regardless of the CSF findings, as our study cannot exclude a possible overlap in CSF/serum ADA ratios or CSF glucose levels between patients with encephalitis and cerebral malaria in other settings. The same applies to epidemiological surveillance pending further studies.

## Conclusion

CSF adenosine deaminase alone was not a useful discriminator between encephalitis and cerebral malaria. However, the CSF/serum ADA ratio was lower in patients with cerebral malaria due to the high levels of ADA in peripheral blood. This ratio should be evaluated in future, larger-scale, investigations into discriminatory factors between encephalitis and cerebral malaria. Although CSF glucose was significantly reduced in malaria patients, the results should be interpreted cautiously and require confirmation in a larger study. It would be preferable to use CSF/serum glucose ratios for future studies. Large-scale prospective studies in areas where *P. falciparum *and for example, Japanese encephalitis viruses, are both endemic are needed to validate our findings in different settings.

## Competing interests

The author(s) declare that they have no competing interests.

## Authors' contributions

SRJ participated in the design of the study, recruitment of patients, collection of samples and data-analysis. SV participated in the laboratory analysis and data analysis. RMA participated in design of the study. ME participated in data-analysis, interpreted the data and wrote the paper.
